# PvGAP: Development of a Globally Applicable, Highly Multiplexed Microhaplotype Amplicon Panel for *Plasmodium vivax*

**DOI:** 10.1093/infdis/jiag270

**Published:** 2026-05-21

**Authors:** Alfred Hubbard, Edwin Solares, Lauren Bradley, Brook Jeang, Delenasaw Yewhalaw, Daniel Janies, Eugenia Lo, Guiyun Yan, Elizabeth Hemming-Schroeder

**Affiliations:** Department of Epidemiology, Johns Hopkins Bloomberg School of Public Health, Baltimore, Maryland, USA; Department of Computer Science and Engineering, UC San Diego, San Diego, California, USA; Department of Population Health & Disease Prevention, UC Irvine Wen School of Population and Public Health, Irvine, California, USA; Department of Population Health & Disease Prevention, UC Irvine Wen School of Population and Public Health, Irvine, California, USA; School of Medical Laboratory Sciences, Jimma University, Jimma, Ethiopia; Tropical and Infectious Diseases Research Center, Jimma University, Jimma, Ethiopia; Department of Bioinformatics and Genomics, University of North Carolina at Charlotte, Charlotte, North Carolina, USA; Department of Microbiology and Immunology, Drexel University, Philadelphia, Pennsylvania, USA; Department of Population Health & Disease Prevention, UC Irvine Wen School of Population and Public Health, Irvine, California, USA; College of Veterinary Medicine and Biomedical Sciences, Colorado State University, Fort Collins, Colorado, USA

**Keywords:** *Plasmodium vivax*, genomic epidemiology, amplicon sequencing, microhaplotypes, molecular surveillance

## Abstract

**Background:**

*Plasmodium vivax* malaria research has yet to fully benefit from advances in genomic surveillance that have revolutionized *P. falciparum* epidemiology. Closing this gap will enable monitoring the spread of drug resistance, classifying infections as local or imported, and distinguishing reinfection, recrudescence, and relapse. To achieve these objectives, microhaplotype marker panels that allow powerful genotyping of polyclonal infections are needed.

**Methods:**

We designed a globally applicable amplicon panel for *P. vivax* (PvGAP), selecting targets based on both genetic diversity and distance to maximize discriminatory capability between geographic regions. We evaluated this panel with field samples from Ethiopia and *in silico* using whole genomes from the MalariaGEN Pv4 database.

**Results:**

PvGAP has 80 high-diversity targets suitable for population genomics and 8 targets of epidemiological interest, such as putative markers of drug resistance. We demonstrate robust amplification with field data and competitive accuracy for relatedness inference in 3 disparate geographic regions.

**Conclusions:**

PvGAP joins existing *P. vivax* panels as a cost-effective and practical option for genomic epidemiology of this neglected disease. It will support drug resistance surveillance and discrimination of local and imported cases, and it may aid in separating reinfection, recrudescence, and relapse in therapeutic efficacy studies, all critical needs of National Malaria Control Programs.

## Teaser

Globally applicable Amplicon Panel for *Plasmodium vivax* is an 88-marker panel for amplicon sequencing of *P. vivax* that enables various genomic epidemiology applications. It is similar to existing panels, but offers potential advantages in terms of flexibility and cost.

*Plasmodium vivax* remains understudied compared to *P. falciparum*, despite the fact that it can cause severe and life-threatening malaria [1] and is endemic in tropical regions throughout the world, including most of Africa [2]. Improving our understanding of *P. vivax* epidemiology is a key step to planning effective antimalarial interventions and ultimately eliminating malaria.

Population genomics is one powerful approach for enhancing our collective understanding of malaria epidemiology. Genomic data from malaria parasites can be used to assess transmission between geographic locations and can support modeling of relapse, recrudescence, and reinfection [3]. However, the application of population genomics to malaria epidemiology is limited by technical constraints and costs. Classical biallelic single nucleotide polymorphism (SNP) assays have low sensitivity to detect multiple parasite strains and parasite diversity within a host [4]. On the other hand, whole genome sequencing data provide comprehensive coverage, but is costly to produce [5] and store [3].

A cost-effective alternative with high sensitivity to detect minority clones is targeted deep sequencing of genetically diverse amplicons [4, 5]. This approach is particularly effective when used to assess multiallelic microhaplotypes, meaning targets that contain 2 or more SNPs. Microhaplotype markers have been shown to provide higher power for relatedness inference than biallelic SNPs, particularly in the case of polyclonal infections [5]. The benefits of microhaplotype panels for understanding genetic relatedness and diversity have already been demonstrated in *P. falciparum* [5, 6].

Several microhaplotype panels have been or soon will be published for *P. vivax*. Particularly noteworthy are the Kleinecke et al. [7] and PvGTSeq [8] panels, which both contain putative markers of drug resistance and numerous high-diversity targets (93 for Kleinecke et al. and 213 for PvGTSeq). A handful of other panels have been developed with smaller sets of microhaplotypes [9–12]. Given that biallelic markers, which comprise most of the Kattenberg et al. [10], Kattenberg et al. [9], and Popkin-Hall et al. [11] panels, are fundamentally limited in the presence of polyclonal infections [5] and the small size of the Rosado et al. [12] panel (11 targets), these panels are likely to have less power for relatedness estimation than the PvGTSeq and Kleinecke et al. panels. They may retain sufficient power in certain settings and for other applications such as estimating the number of unique strains in an infection (ie, the complexity of infection).

This paper describes the design and validation of a *Plasmodium vivax* globally applicable amplicon panel (PvGAP) that is similar in scope to the Kleinecke et al. [7] and PvGTSeq [8] panels. PvGAP consists of 80 high-diversity microhaplotype markers, 7 putative drug resistance markers, and a section of *pvdbp* (*P. vivax* Duffy binding protein). The associated library preparation protocol is relatively low cost and uses nonproprietary reagents. Therefore, PvGAP is a powerful and flexible addition to the *P. vivax* population genomics toolkit that should suit a variety of epidemiological applications.

## METHODS

### Panel Design

Whole genome sequence data from 198 *P. vivax* isolates from 8 countries were downloaded from NCBI (Table 1). Reads were aligned to the PvP01 reference genome [22], and variants were called using standard bioinformatic pipelines. Candidate loci were identified using a sliding-window method adapted from Tessema et al. [5]. Genomic windows were filtered to remove repetitive regions, indels, and loci lacking within-country polymorphism. Remaining windows were evaluated based on within-country nucleotide diversity (π) and population differentiation (F_ST_) among countries. Windows with high-diversity and/or strong genetic structuring were prioritized across chromosomes, yielding 278 candidate targets distributed across the genome. Detailed bioinformatic methods, filtering criteria, and marker selection procedures are provided in Supplementary Text 1.

**Table 1.** *P. vivax* Genomes Used in Panel Design

Country	No. of Genomes	Reference
Cambodia	31	Parobek et al. [13]
China	28	Chen et al. [14], Shen et al. [15]
Colombia	8	Winter et al. [16]
Ethiopia	33	Auburn et al. [17] Lo et al. [18]
Madagascar	9	Menard et al. [19]
Malaysia	30	Auburn et al. [20]
Panama	29	Accession: PRJNA655141
Peru	30	Cowell et al. [21]

These targets were submitted to GTseek LLC (Twin Falls, ID, USA) for multiplex primer design optimized to minimize primer cross talk [23]. After initial sequencing tests, poorly performing targets were removed, resulting in a final set of 80 loci. Eight additional loci targeting *pvdbp* and putative drug resistance markers were added to the panel (Table 2), resulting in a final panel of 88 loci (Supplementary Data 1).

**Table 2.** *P. Vivax* Genes of Particular Epidemiological Interest

Gene Name	Gene ID	Chromosome	Start^a^	End^a^
*pvdbp*	PVP01_0623800	PvP01_06_v1	983467	983623
*pvcrt*	PVP01_0109300	PvP01_01_v1	442085	442281
*pvdhfr*	PVP01_0526600	PvP01_05_v1	1077441	1077602
*pvdhps*	PVP01_1429500	PvP01_14_v1	1270726	1270916
*pvdhps*	PVP01_1429500	PvP01_14_v1	1270343	1270530
*pvk13*	PVP01_1211100	PvP01_12_v1	485037	485238
*pvk13*	PVP01_1211100	PvP01_12_v1	486524	486684
*pvmdr1*	PVP01_1010900	PvP01_10_v1	479798	479986

^a^Start and end coordinates are zero-based.

### Panel Evaluation With Field Samples

Two sets of field samples were used to evaluate sequencing performance of the panel. First, 10 high-parasitemia samples (6 dried blood spot [DBS] and 4 whole blood samples) were processed using multiple laboratory workflows to assess how different DNA extraction methods (chelex/saponin vs spin column kit) and enrichment strategies (selective whole genome amplification or SWGA vs targeted preamplification) affected sequencing outcomes. A detailed description of these procedures is provided in Supplementary Text 2. Second, to assess how parasite density influences sequencing output, serial dilutions were generated from 5 high-parasitemia DBS samples to mimic realistic low-parasitemia infections. The procedures and results of this analysis are described in the main text below.

#### Sample Collection

The samples used for the serial dilutions were gathered as part of a sub-Saharan Africa International Center for Excellence for Malaria Research project [24, 25]. They were collected as DBS through both passive case detection and community cross-sectional surveys from 2 sites in Southwestern Ethiopia. Details on sample collection may be found in Getachew et al. [26].

#### Library Preparation and Sequencing

DNA was extracted from DBS using the Monarch Spin gDNA extraction kit (New England Biolabs). Briefly, a 6-mm DBS punch was lysed and genomic DNA purified following the manufacturer's protocol with minor modifications, with DNA eluted in 35 µL of TE (Tris EDTA) buffer. Parasite DNA concentration was quantified by quantitative PCR using primers and probes described by Lee et al. [27] and a standard curve generated from serial dilutions of the *P. vivax* Sal I Sau3AI gDNA library (MRA-40, BEI Resources). Additional details of DNA extraction and quantification are provided in Supplementary Text 1.

Following quantification, samples were diluted to target parasite DNA concentrations of 1000, 500, 250, 100, 50, and 10 copies/µL. To generate serial dilutions that more closely mimic natural infections with low parasitemia (ie, similar ratios of human to parasite gDNA), gDNA was extracted from uninfected human DBS following the same purification protocol and used as the diluent. Because a gDNA library was used as the quantification standard due to limited availability of parasite material, copy number estimates cannot be directly translated to parasitemia. However, assuming an approximate extraction efficiency of 30% and an input volume of 10 µL of whole blood per 6 mm DBS punch, a measured concentration of 10 parasite copies/µL of eluate would correspond to approximately 11.7 parasites/µL of whole blood. This estimate should be interpreted as an approximation given variability in DBS blood volume and extraction recovery.

The library preparation protocol is described in detail in Supplementary Text 3. In brief, it consists of SWGA, adapted from Oyola et al. [28] and Cowell et al. [29], to increase the density of parasite DNA; an adapted version of the GT-seq PCR protocol to affix primers (see Campbell et al. [23] for the original technique and LaVerriere et al. [6] for a previous application to *P. falciparum*); and application of Nate's Plates kits (GTseek LLC) to affix dual indexing tags and normalize sequence quantity across samples. Various quality control (QC) assays are performed after each step and on the final libraries, as described in Supplementary Text 3. Sequencing was performed on an Illumina MiSeq, using the Reagent Kit v2 500 cycles kit in paired-end mode with a 10% PhiX spike-in. Samples were amplified and sequenced in triplicate.

#### Bioinformatics Analysis

The malaria amplicon pipeline published alongside LaVerriere et al. [6] was used to extract and filter microhaplotypes, using primer and reference files appropriate for our *P. vivax* panel (available at https://doi.org/10.5281/zenodo.20450439). This pipeline uses the Divisive Amplicon Denoising Algorithm (DADA2 [30]) as the core method for identifying and filtering amplicons. The pipeline then applies a handful of postprocessing steps to remove likely sequencing artifacts and chimeras.

Amplicons with fewer than 5 reads or a within-sample allele frequency (WSAF) below 1% were filtered out. Lin's concordance correlation coefficient was computed among replicate pairs with the DescTools R package [31].

### Panel Evaluation With Paneljudge and MalariaGEN Data

#### Data Preparation

To assess the utility of the panel for population genetics and genomic epidemiology, whole genome sequences from the MalariaGEN Pv4 project [32] were downloaded and virtual microhaplotypes were extracted. Variants that failed the QC process of the MalariaGEN authors (FILTER = “PASS” in the variant call format) were excluded. Samples with an *F*_WS_ below 0.95 were removed, as these are considered to be polyclonal [33]. Finally, samples from longitudinal studies and returning travelers were removed. From the remaining samples, 3 populations were selected based on sample size and representation of distinct regions: Cambodia and Vietnam in 2015 and 2016 (*n* = 72); Brazil, Colombia, and Peru in 2013 and 2014 (*n* = 43); and Ethiopia in 2013 (*n* = 49).

The remaining variants were filtered to the genomic regions represented in PvGAP using bcftools view [34]. Haplotype sequences were obtained for each sample and locus with bcftools consensus [34], using the PvP01 reference genome [22]. To enable comparison with other amplicon panels, the diversity targets of the Kleinecke et al. [7] and PvGTSeq [8] panels were extracted with the same methods. Other *P. vivax* microhaplotype panels were excluded from this analysis because they either lack diverse microhaplotype targets [9–11] or are considerably smaller than the 3 panels included in the evaluation [12].

#### Paneljudge Analysis

For each population, the allele frequencies of all panel targets were calculated with the Dcifer R package [35]. Cardinality (the number of unique alleles at a locus) and effective cardinality (the number of unique alleles adjusted for their frequency; see Taylor et al. [36]) were calculated from these frequencies with the paneljudge R package (https://github.com/aimeertaylor/paneljudge; v0.0.0.9). The paneljudge package was also used to simulate pairwise relatedness estimates for each panel under various true values of *r*: 0.01, 0.05, 0.1, 0.15, 0.2, 0.25, 0.5, 0.75, and 0.99. These simulations were performed with 100 sample pairs drawn from each population, 100 bootstrap replicates, and a switch-rate parameter value of 5.

### Multiplexing Capacity

To estimate multiplexing capacity, we used locus-level read counts from the sequencing dataset generated in this study. Total on-target reads per sample and locus-specific read proportions were calculated, and relative sample efficiencies were derived to capture uneven sample representation in multiplexed libraries. We then used empirically parameterized Monte Carlo resampling to model expected locus coverage across Illumina sequencing kits with different read outputs. For each kit and candidate multiplex level, we assumed that 30% of reads would be lost (10% due to PhiX and 20% due to off-target amplification). We repeated the resampling procedure 100 times. Samples were considered to pass if at least 75% of loci achieved ≥10 reads, and recommended multiplexing levels were defined as the largest sample number for which ≥80% of samples were predicted to meet this criterion. Additional details are provided in Supplementary Text 5.

## RESULTS

### Panel Characteristics

**Figure 1.**
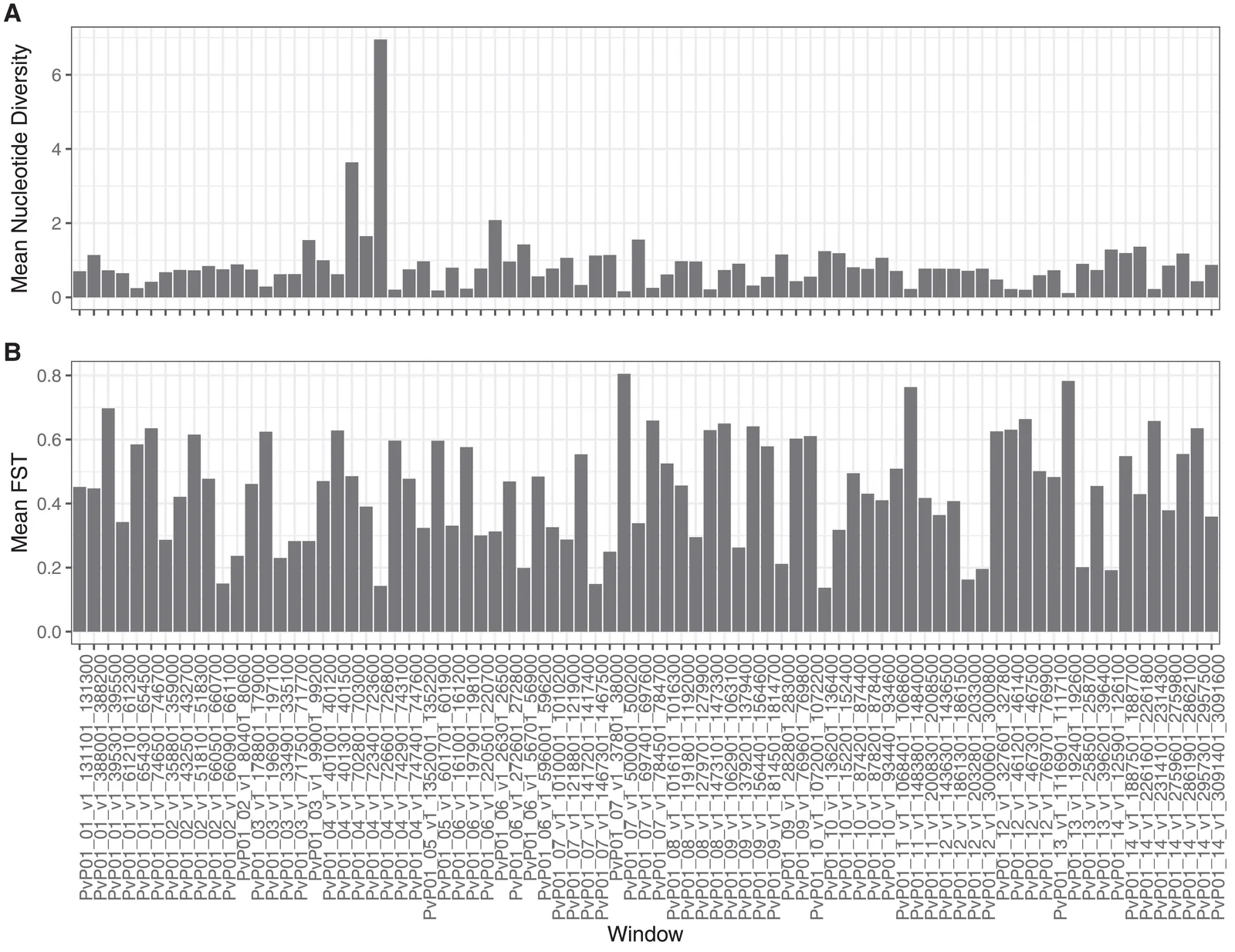
Mean nucleotide diversity (*A*) and mean F_ST_ (*B*) for the genome windows selected for inclusion in the final panel.

Windows selected during panel design were chosen based on high nucleotide diversity and/or high F_ST_. Consistent with these criteria, all windows included in the final marker set exhibited high mean nucleotide diversity (median = 0.7552; interquartile range [IQR] = 0.5555–0.9803; Figure 1*A*) and/or high mean F_ST_ (median = 0.4555, IQR = 0.31–0.5964; Figure 1*B*) in the genome set used for panel design.

### Panel Evaluation With Field Samples

Detailed results for the samples used for laboratory workflow optimization are described in Supplementary Text 2. In brief, both extraction methods performed similarly, and differences between enrichment strategies were modest. SWGA yielded a slightly higher proportion of on-target reads, whereas targeted preamplification resulted in slightly more even coverage across markers. SWGA and the chelex/saponin method were selected for the final protocol.

**Figure 2.**
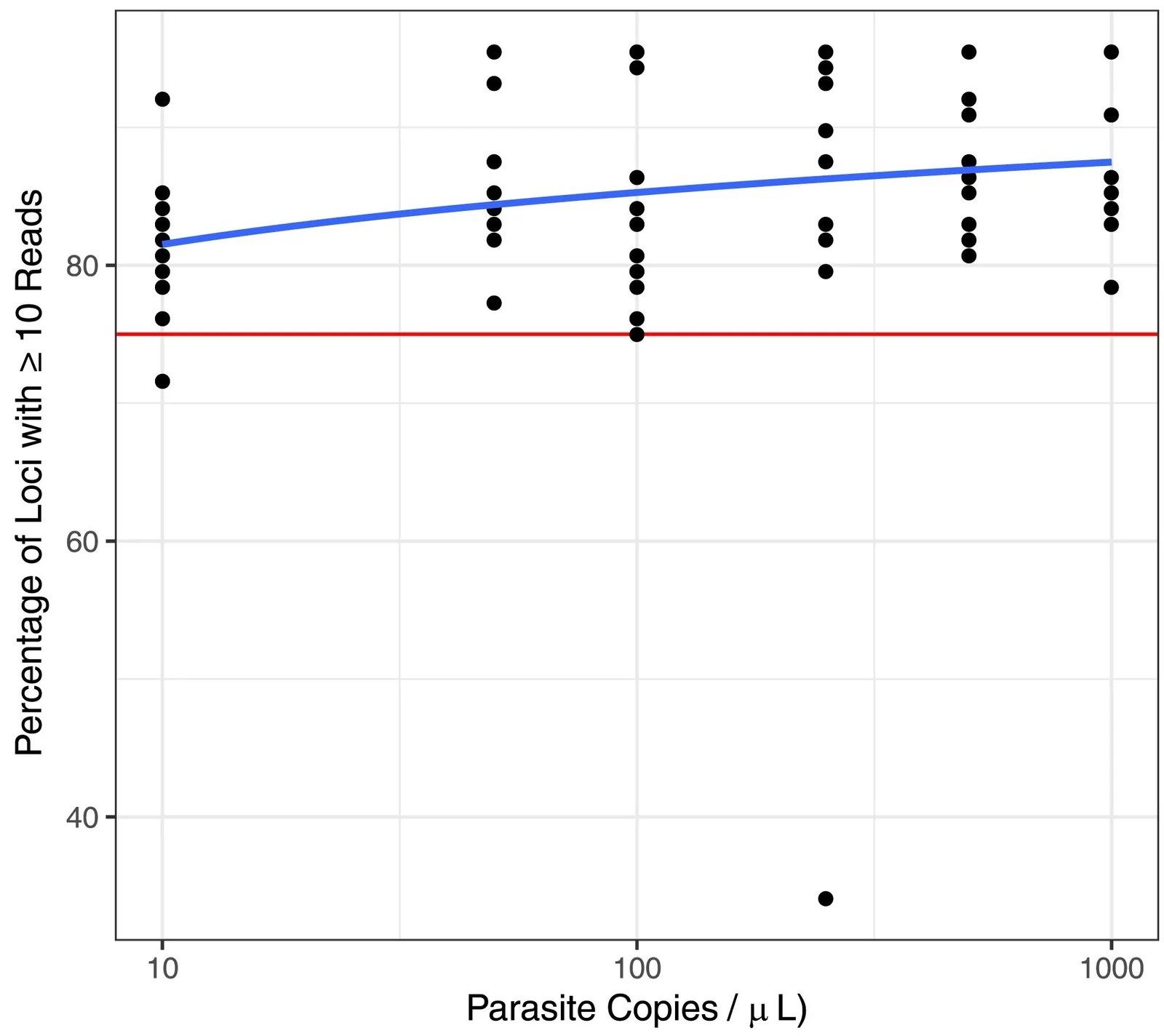
Scatterplot showing the relationship between parasite copy number and the percentage of loci with 10 or more reads (indicating successful amplification) for each replicate of the serial dilutions. The *x*-axis is log_10_ scaled. The curved blue line is a simple linear regression. The horizontal red line indicates 75% successful amplification, a common threshold for resequencing a sample.

In the serial dilution experiments, mean read counts across replicates (Supplementary Figure 1) indicate that overall amplification was satisfactory (median read depth per marker of 82; IQR = 22–391). A few loci exhibited lower amplification efficiency. Notably, these loci performed well in prior experiments (Supplementary Text 2), suggesting that this reduced performance is unlikely to represent intrinsic issues with the primers. Despite the reduced sequencing output at these loci, the proportion of targets with a minimum of 10 reads remained above 75% among all dilutions (Figure 2), suggesting the SWGA protocol enables reliable amplification in low parasite copy number samples. Moreover, off-target reads were low, with a median of 79.75% on-target reads per sample (IQR = 71.68–80.85%).

Detection of minority alleles across replicates was inconsistent, though not strongly affected by parasite copy number (Supplementary Figure 2). Concordance among replicate pairs ranged widely, from nearly 0 to 1 when computed only on alleles present in both replicates of the pair (Supplementary Figure 3). Notably, concordance varied substantially among samples, possibly reflecting the presence of minor parasite strains at low densities or variation introduced during dilution preparation. When alleles only found in one replicate were included, concordance ranged from 0 to 0.6.

### Panel Evaluation With Paneljudge and MalariaGEN Data

**Figure 3.**
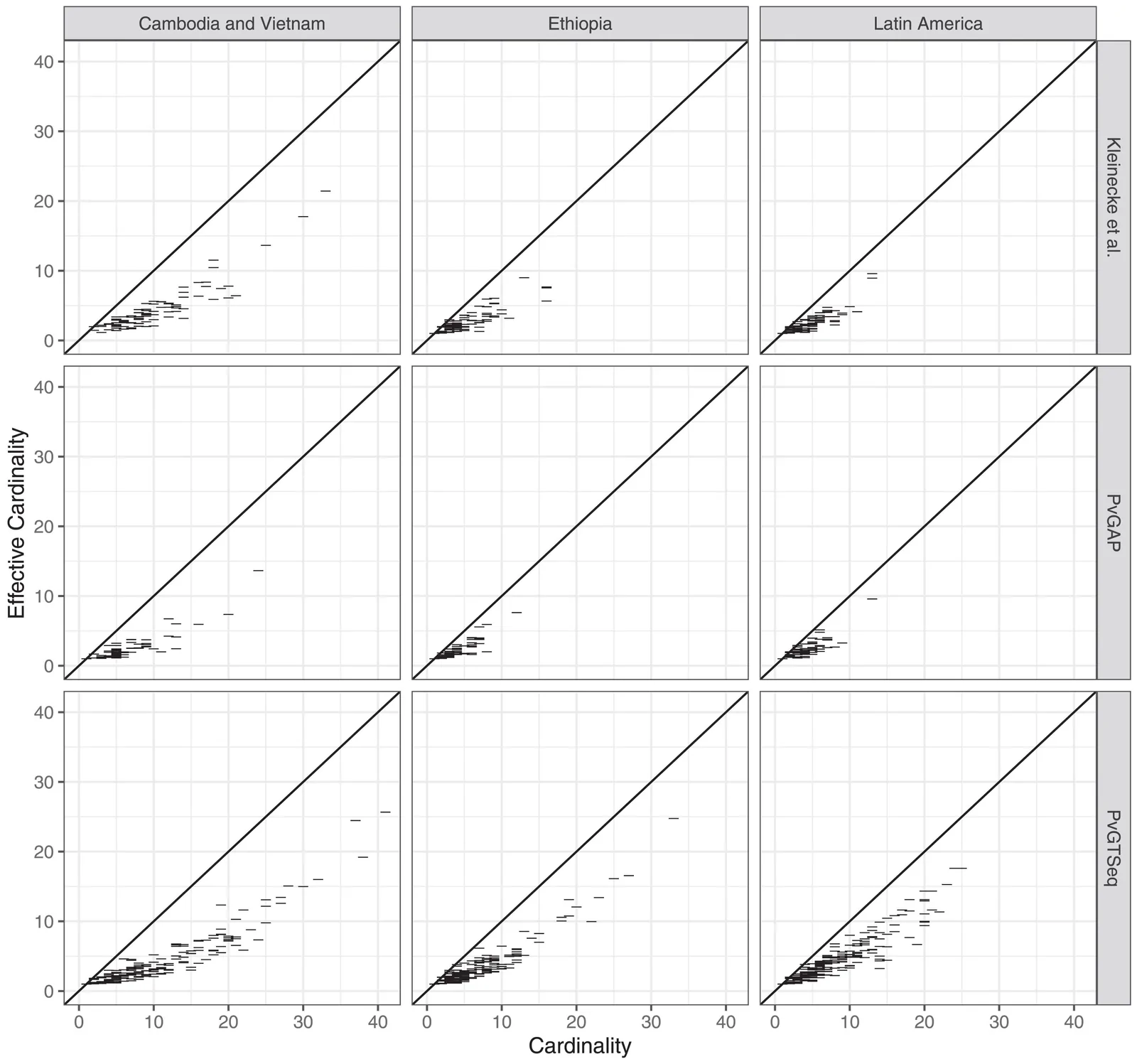
Cardinality (the number of unique alleles) versus effective cardinality (the number of unique alleles adjusted for allele frequency) for each marker (horizontal ticks indicate markers), based on the MalariaGEN data. The diagonal line shows *y* = *x*, which is the theoretical maximum of effective cardinality. The facets show the markers separately for each panel and population.

Comparison of the PvGAP panel with the panels from Kleinecke et al. [7] and PvGTseq revealed that cardinalities (ie, the number of unique alleles at each target) followed a similar pattern, with most markers showing a cardinality below 10 in most populations (Figure 3). Larger panels tended to have more high cardinality markers, though; PvGTSeq contains several markers with a cardinality above 20. In all panels, target effective cardinalities were fairly close to their theoretical maximum, the cardinality, suggesting the targets have sufficient allelic evenness to be informative for relatedness analyses (Figure 3).

**Figure 4.**
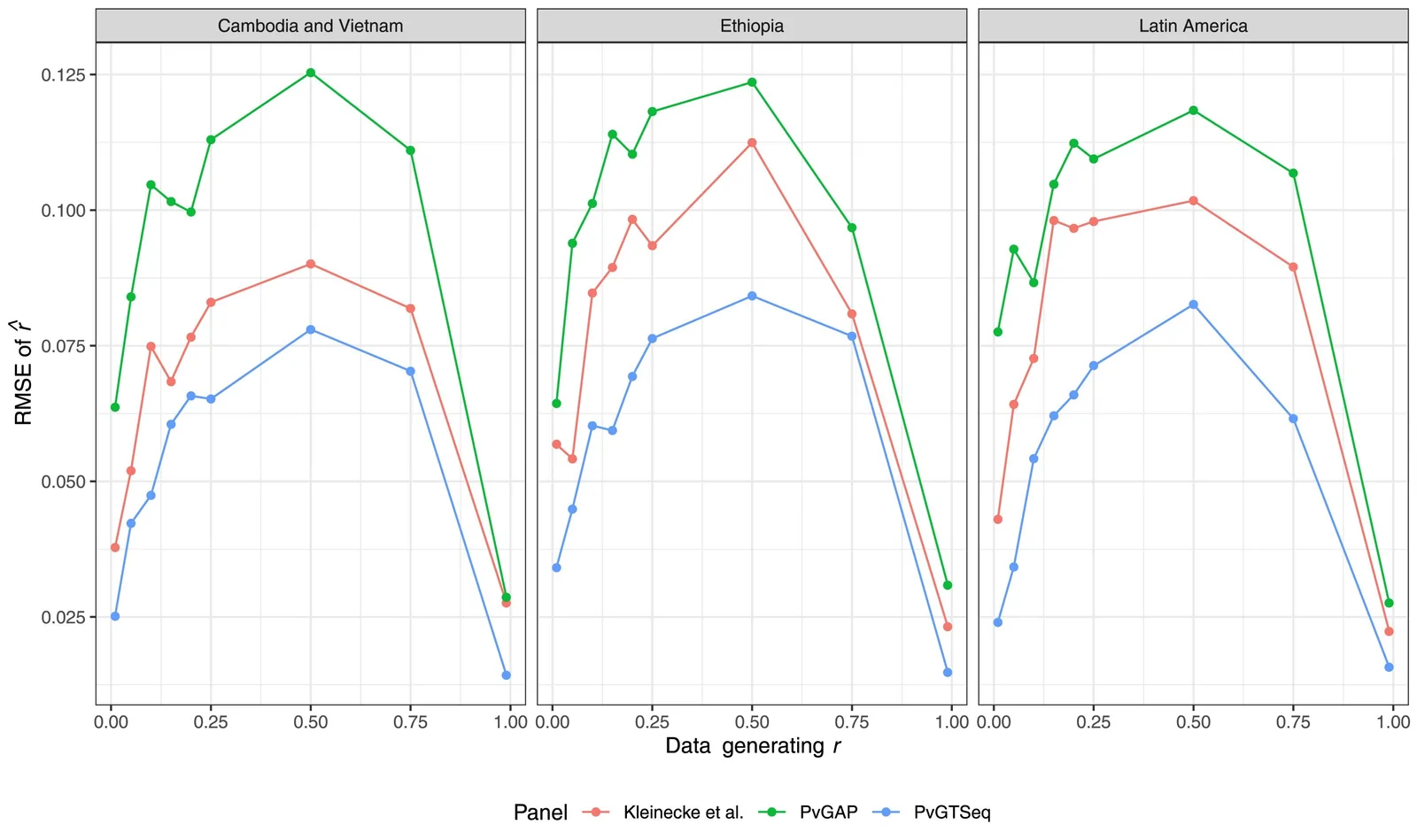
The RMSE of $\hat{r}$ for each data-generating *r* value, as simulated by paneljudge. Facets distinguish results for different populations. Line and point colors indicate different marker panels.

The paneljudge simulations indicate all 3 panels have substantial power for relatedness estimation. The root mean square errors (RMSEs) of $\hat{r}\;$were 0.0625–0.125 for data-generating *r* values between 0.15 and 0.75 and lower for data-generating *r* values near 0 or 1 (Figure 4). Similarly, the 95% confidence interval widths of these $\hat{r}$ estimates tend to be wider (0.2–0.6) for intermediate data-generating *r* values and narrower near 0 and 1 (Supplementary Figure 4). As expected from the number of diversity markers, PvGTSeq consistently yielded the lowest RMSE, followed by the Kleinecke et al. panel, followed by PvGAP. The differences are larger for intermediate data-generating *r* values and negligible for a data-generating *r* value of 0.99, indicating all panels are equally equipped to identify clonal pairs. PvGTSeq and PvGAP exhibit little difference in performance between geographic regions; the Kleinecke et al. panel exhibits slightly better performance in Southeast Asia than Ethiopia or Latin America. Notably, in all instances, for all panels, the 95% confidence interval widths are substantially <1.

### Multiplexing Capacity and Sequencing Cost

Based on sample-to-sample unevenness, on-target read percentages, and locus-specific read counts observed in our dataset, we simulated multiplexing performance across Illumina sequencing kits to estimate feasible multiplexing levels and associated costs. For the MiSeq v2 platform, the recommended multiplexing level is 136 samples per run, corresponding to an estimated sequencing cost of 13.24 USD per sample and a combined library preparation and sequencing cost of 28.24 USD per sample. Additional details and results for other sequencing kits are described in Supplementary Text 5.

## DISCUSSION

PvGAP represents a cost-effective approach to targeted sequencing of *P. vivax* microhaplotypes for genomic epidemiology applications in varied geographic locations. The panel consists of 88 loci, including 80 selected for population genetic and relationship inference and 8 within genes of epidemiological interest (eg, potential drug resistance/tolerance markers). Our assay consistently yields reliable sequencing depth and breadth of coverage, even in the case of samples with low parasite copy numbers (10 parasites/μL), and does not rely on proprietary reagents. *In silico* evaluation with MalariaGEN data and paneljudge demonstrates that PvGAP contains sufficient marker diversity for relatedness estimation in a variety of geographic regions, albeit with lower accuracy than larger panels.

PvGAP displayed strong sequencing performance in terms of proportion of successfully amplified loci (all serial dilutions achieved >75% loci with ≥10× coverage) and percentage of on-target reads (median 79.75%). The former metric compares favorably to similar assays (Kleinecke et al. and PvGTseq). Multiplexing levels and on-target read percentages were not consistently reported by Kleinecke et al. [7] and Manrique-Valverde et al. [8], limiting direct comparison of sequencing efficiency and scalability of assays, particularly for DBS-derived samples. Regarding reproducibility across technical replicates, major allele detection was highly reproducible, but concordance of WSAFs varied among samples. This result may stem from stochastic detection of low-frequency minority clones, but this cannot be confirmed as these are field samples with unknown true WSAFs. Future benchmarking with synthetic mixtures could clarify interpretation of WSAF estimates and the need for further methodological refinements, if any.

In terms of relatedness estimation, the paneljudge analysis showed good performance of PvGAP, albeit slightly worse than similar, larger panels. All 3 panels evaluated (PvGAP, PvGTSeq, and Kleinecke et al.) perform well when the true relatedness is high or low and somewhat worse when the true relatedness is intermediate (between 0.15 and 0.75). Although PvGTSeq consistently yielded the lowest RMSE, and PvGAP the highest RMSE (ranging from 0.028 to 0.125 depending on relatedness level and region), the importance of reducing RMSE should be evaluated in light of study objectives, budgetary considerations, and the biological limits inherent to malaria transmission inference. At present, the degree of precision required to reliably distinguish specific transmission events remains incompletely defined and likely varies by epidemiological setting and sampling design. Further empirical and simulation-based work is needed to determine the added value of sequencing larger, more costly panels that enable more accurate relatedness estimates.

Regarding cost, PvGAP is an affordable and flexible option. Library preparation for PvGAP is approximately 13 USD per sample (excluding primer costs; see below), compared to roughly 10–20 USD (converted from AUD) for the Kleinecke et al. [7] assay, depending on whether the PCR reaction volume is halved. Once sequencing is included, we estimate a combined cost of approximately 28 USD per sample for PvGAP in a MiSeq v2 run. Manrique-Valverde et al. [8] report an estimated cost of approximately 39 USD per sample for library preparation and sequencing for PvGTSeq. Primer synthesis constitutes an upfront cost that scales with the number of targets. We estimate each locus requires approximately 24 USD in primer synthesis. Assuming the same cost for PvGTSeq, initial investment increases by several thousand USD. Finally, like the PvGTseq panel, the PvGAP primers have been designed to minimize cross talk during multiplexing and library preparation by relying on standard amplicon-based methods rather than vendor-specific capture chemistries. In contrast, the Kleinecke et al. assay relies on Integrated DNA Technologies' proprietary rhAmpSeq methodology [7], which may influence cost structure, supply-chain flexibility, and implementation considerations.

PvGAP is a moderately sized panel for *P. vivax* epidemiology that provides a practical balance of feasibility and resolution for relatedness inference. In comparison to similar, previously published panels, PvGAP offers competitive cost, slightly worse accuracy for relatedness estimation due to fewer markers, and a flexible laboratory protocol that can be adapted to a variety of settings. By substantially expanding the array of available panels, PvGAP helps ensure that the appropriate tool will be available for every application of genomic epidemiology to *vivax* malaria.

## Supplementary Material

jiag270_Supplementary_Data

## Supplementary Data

Supplementary materials are available at *The Journal of Infectious Diseases* online (http://jid.oxfordjournals.org/). Supplementary materials consist of data provided by the author that are published to benefit the reader. The posted materials are not copyedited. The contents of all supplementary data are the sole responsibility of the authors. Questions or messages regarding errors should be addressed to the author.
